# Systematic tissue collection during clinical breast biopsy is feasible, safe and enables high-content translational analyses

**DOI:** 10.1038/s41698-021-00224-w

**Published:** 2021-09-21

**Authors:** Siang-Boon Koh, Brian N. Dontchos, Veerle Bossuyt, Christine Edmonds, Simona Cristea, Nsan Melkonjan, Lindsey Mortensen, Annie Ma, Kassidy Beyerlin, Elyssa Denault, Elizabeth Niehoff, Taghreed Hirz, David B. Sykes, Franziska Michor, Michelle Specht, Constance Lehman, Leif W. Ellisen, Laura M. Spring

**Affiliations:** 1 MGH Cancer Center, Massachusetts General Hospital, Boston, MA USA; 2 Harvard Medical School, Boston, MA USA; 3 Department of Data Science, Dana-Farber Cancer Institute, Boston, MA USA; 4 Center for Regenerative Medicine, Massachusetts General Hospital, Boston, MA USA; 5 Harvard Stem Cell Institute, Cambridge, MA USA; 6 Department of Stem Cell and Regenerative Biology, Harvard University, Cambridge, MA USA; 7 Ludwig Center at Harvard, Boston, MA USA; 8 The Broad Institute of MIT and Harvard, Cambridge, MA USA; 9 Center for Cancer Evolution, Dana-Farber Cancer Institute, Boston, MA USA

**Keywords:** Breast cancer, Tumour heterogeneity

## Abstract

Systematic collection of fresh tissues for research at the time of diagnostic image-guided breast biopsy has the potential to fuel a wide variety of innovative studies. Here we report the initial experience, including safety, feasibility, and laboratory proof-of-principle, with the collection and analysis of research specimens obtained via breast core needle biopsy immediately following routine clinical biopsy at a single institution over a 14-month period. Patients underwent one or two additional core biopsies following collection of all necessary clinical specimens. In total, 395 patients were approached and 270 consented to the research study, yielding a 68.4% consent rate. Among consenting patients, 238 lesions were biopsied for research, resulting in 446 research specimens collected. No immediate complications were observed. Representative research core specimens showed high diagnostic concordance with clinical core biopsies. Flow cytometry demonstrated consistent recovery of hundreds to thousands of viable cells per research core. Among a group of HER2 + tumor research specimens, HER2 assessment by flow cytometry correlated highly with immunohistochemistry (IHC) staining, and in addition revealed extensive inter- and intra-tumoral variation in HER2 levels of potential clinical relevance. Suitability for single-cell transcriptomic analysis was demonstrated for a triple-negative tumor core biopsy, revealing substantial cellular diversity in the tumor immune microenvironment, including a prognostically relevant T cell subpopulation. Thus, collection of fresh tissues for research purposes at the time of diagnostic breast biopsy is safe, feasible and efficient, and may provide a high-yield mechanism to generate a rich tissue repository for a wide variety of cross-disciplinary research.

Emerging research on the dynamic and complex nature of breast cancer and its precursors has propelled precision medicine to the forefront of breast cancer care. The use of clinical molecular assays is increasing in the early breast cancer setting, and multiple targeted therapies are now being applied in the metastatic setting based on genomic testing^1–4^. A greater understanding of breast cancer biology and evolution holds much promise for improving outcomes. Increasing access to fresh, invasive, and noninvasive breast lesions is crucial to accelerating such research. For example, such tissues are essential to enable studies of early cancer pathogenesis, through analysis of non-cancerous “high-risk lesions” including atypical ductal hyperplasia and lobular neoplasia. Furthermore, cancer-positive biopsies allow assessment of molecular features associated with treatment response in patients destined to undergo neoadjuvant therapy. The analysis of such specimens can inform the entire spectrum of questions regarding mechanisms of breast cancer risk, pathogenesis, disease progression, treatment response, and disease outcomes.

A potentially efficient approach for systematic collection of fresh breast tissue for research purposes is to perform one or more additional needle passes immediately following diagnostic image-guided biopsy. Typical clinical protocols for the collection of diagnostic specimens employ a core biopsy needle ranging from 9 to 18 gauge under image guidance. While the amount of tissue collected in these biopsies is relatively small, new technologies for the analysis of entire genomes and transcriptomes at the level of individual cells have dramatically increased the type and amount of data that can be gleaned from such specimens^5^.

Nevertheless, multiple aspects related to the feasibility of such an approach need to be addressed prior to widespread implementation. Practical issues include the method and timing of the informed patient consent, logistical issues related to the clinical workflow, and safety issues associated with additional research sampling. Furthermore, it is critical to assess the quality of research specimens obtained in this manner and the extent to which they are representative of the clinical diagnostic material. Finally, laboratory proof-of-principle is required, demonstrating the ability to process and effectively analyze such specimens for a variety of research applications.

We sought to address these issues by initiating a systematic program for collection of the research tissue at the time of diagnostic breast biopsy with the informed patient consent. The initial goals of the study were assessment of feasibility and suitability of the tissue for research applications. Here we describe the initial experience with the collection of more than 400 core biopsy breast tissue research specimens obtained immediately following routine clinical image-guided breast biopsy at a single institution over a 14-month period.

## Feasibility, safety, and cohort collection

**Fig. 1:**
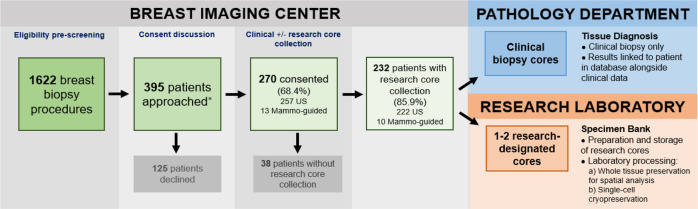
Systematic acquisition of research core biopsy specimens is feasible. Workflow for multi-departmental effort in collecting clinical and research biopsy cores from eligible and consented patients. Between January 2019 and March 2020, a total of 232 consenting patients whose lesions were biopsied for research were successfully enrolled, where 3–5 clinical cores per lesion were sent to pathology for diagnostic evaluation, and one or two research-designated cores per lesion were sent to the research laboratory for research core specimen banking. Clinical and pathologic data were linked with the patient in a REDCap database for future multidisciplinary research. *Initially, only patients undergoing ultrasound (US)-guided core biopsy were approached and consented. Over time, a limited number of patients undergoing mammographic-guided biopsy were approached and consented.

A total of 1622 clinical breast biopsy procedures (ultrasound-guided or mammographic-guided) were performed at our institution between January 2019 and March 2020. Among those undergoing a clinical core needle breast biopsy, 395 patients were approached and 270 consented to the study for the research core collection, yielding a 68.4% consent rate. Of the 270 patients who consented to the program, we succeeded in collecting research biopsy cores from 232 (85.9%) patients for the research (mean age 50.6, range 18–88). For the 14.1% (38/270) of consenting patients whose research cores were not taken, causes included insufficient tissue, extra core collection being ultimately deemed too risky, the procedure being converted to a cyst aspiration, and the patient opting to postpone the biopsy procedure following the consent. The operational workflow for this program is detailed in Fig. 1. No immediate complications, including ongoing bleeding, vasovagal reaction, or pneumothorax, were observed in the 232 patients undergoing both the clinical and research breast biopsies. During the same period, zero complications were reported among the non-research procedures (1622 breast biopsy procedures, ultrasound (US)-guided or mammographic-guided).

Among the 232 consenting patients from whom research cores were collected, a total of 446 research cores were obtained (most patients had the two research cores collected from a single lesion). Of these 232 patients, 222 patients underwent biopsy by US guidance, among whom 216 patients underwent research core biopsy of one lesion, and six patients underwent research core biopsy of two lesions (for a total of 228 lesions biopsied under US-guidance for research, with one or two cores per lesion). Collectively, 429 research cores were collected from 222 consenting patients undergoing US-guided biopsy. Based on gross laboratory assessment, ~ 10% of these research cores were considered small (< 2.5 mm), though most of them sank when placed in an aqueous solution, indicating acceptable levels of non-fat cellularity. The remaining ten patients underwent mammographic-guided procedures, yielding research cores that were typically large but fatty, suggesting low cellularity or the predominant presence of adipose cells.

## Pathology results and invasive disease management

The most common primary pathologies of the lesions biopsied for research were benign (44.5%), invasive carcinoma (42.0%), and high-risk lesions (8.4%), as demonstrated in Table 1. The high rate of invasive carcinoma likely reflects our initial limiting of research biopsies to those with a radiographically evident mass measuring at least 0.5 cm. Among the lesions biopsied for research revealing invasive breast carcinoma (N = 98), the majority were hormone receptor-positive (HR +), human epidermal growth factor receptor 2-negative (HER2 –) breast cancer (71.5%), followed by HER2 + breast cancer (16.3%), and TNBC (12.2%). Among consenting the patients with research core biopsy specimen collection that were diagnosed with invasive breast carcinoma (N = 94), 55 patients (58.5%) subsequently underwent upfront surgery, 31 patients (33.0%) received neoadjuvant therapy, seven patients (7.4%) received systemic therapy only due to metastatic disease, concurrent cancer, or comorbidities, and one patient (1.1%) received subsequent care outside the system with outcomes unknown.

**Table 1:** Primary pathology for lesions biopsied for research among patients consented to the breast biopsy project.

Primary Pathology Description	# of lesions	% of N = 238
**Invasive carcinoma**	100	42.0
*Mammary*		
Invasive ductal carcinoma (IDC)	81	34.0
Invasive lobular carcinoma (ILC)	8	3.4
Invasive mammary carcinoma	9	3.8
*Other*		
Metastatic carcinoma^a^	2	0.8
**In situ carcinoma**	12	5.0
Ductal carcinoma in situ	11	4.6
Papillary carcinoma in situ	1	0.4
**High-risk lesion**	20	8.4
Atypical ductal hyperplasia (ADH)	10	4.2
Sclerosing/papillary lesion with focal atypia	1	0.4
Atypical lobular hyperplasia (ALH)	2	0.8
Flat epithelial atypia (FEA)	2	0.8
Radial scar/complex sclerosing lesion	5	2.1
**Benign**	106	44.5
**Total**	**238 biopsies**	

^a^One serous ovarian carcinoma and one anaplastic thyroid carcinoma.

## Concordance assessment and analytical utility

**Fig. 2:**
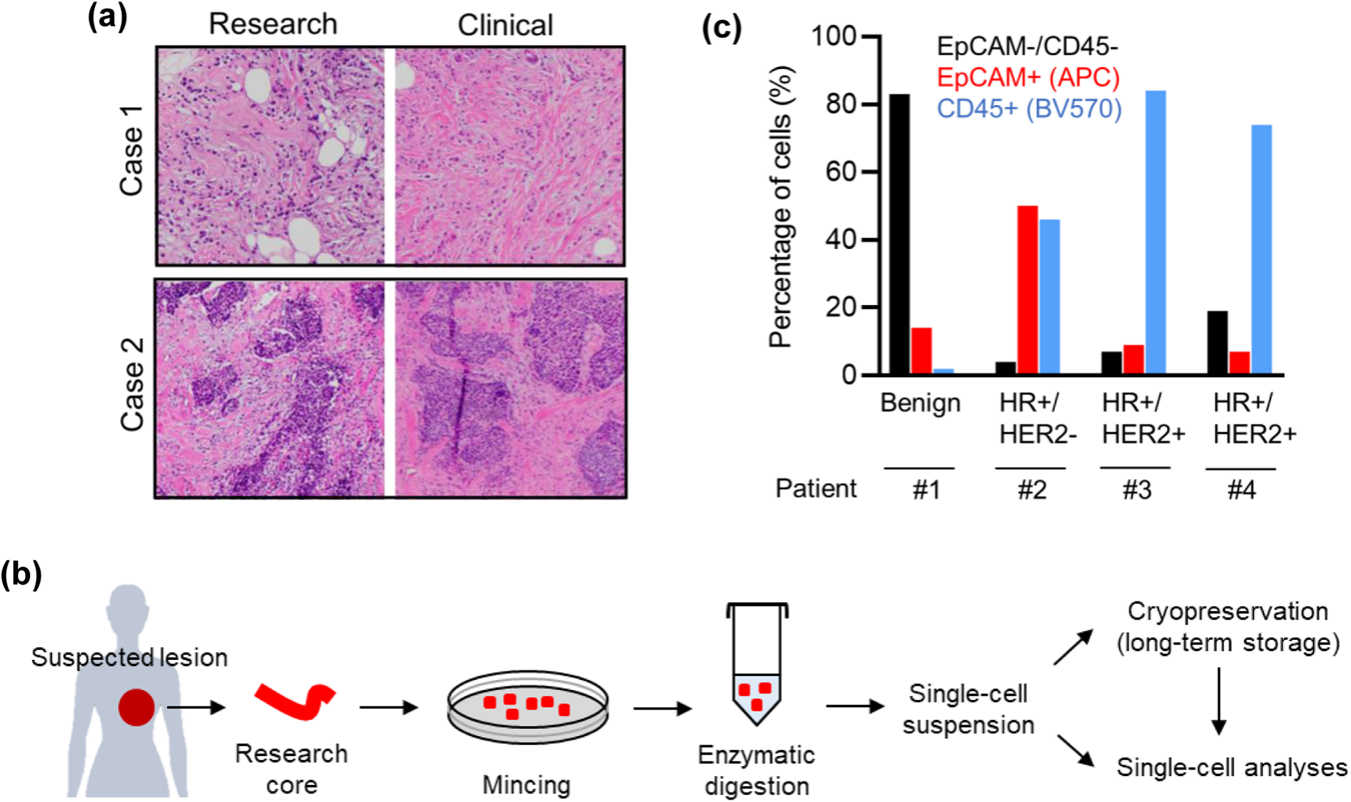
Research core biopsy specimens are histologically concordant with clinical specimens and show expected cell subsets. **a** Histologic concordance of clinical and research core biopsy specimens. H&E staining of representative research core specimens (left) and the clinical core specimens (right), showing grade 2 invasive lobular carcinoma (Case 1; 20x magnification) and grade 2 invasive ductal carcinoma (Case 2, 10x magnification). **b** Workflow for the long-term storage and processing of fresh core biopsy specimens for multiple experimental applications. **c** Cell subsets identified in research core biopsies. Single-cell suspensions were generated from each research core, stained and sorted by flow cytometry, using EpCAM- and CD45-directed antibodies as markers of epithelial and immune cells, respectively.

We next sought to assess the general concordance between clinical and research core biopsy specimens. Using orthogonal approaches, we tested a total of 25 research specimens for diagnostic concordance and analytical utility. First, a randomly selected subset of research core biopsy specimens (10) was fixed, embedded, sectioned, and stained for direct comparison with the corresponding clinical histologic specimen by a pathologist. Overall, 9 of 10 research core biopsy specimens had concordant diagnoses with the clinical specimens, and tumor grade and cellularity were largely similar among invasive cases (Fig. 2a and Supplementary Table 1). For the one research core biopsy that was indeterminant, the clinical specimen showed invasive ductal carcinoma while the research specimen had insufficient tissue for diagnosis.

Among the most promising research applications for such core needle biopsy specimens are single-cell analyses^5–9^. To assess the suitability of our research core biopsy specimens for such analyses, we next developed a protocol for brief mechanical and enzymatic digestion, long-term cryopreservation of cell suspension, antibody staining, and analysis by flow cytometry (Fig. 2b). This approach yielded hundreds to thousands of viable cells post-cryopreservation from a single core needle biopsy (mean = 3132 viable cells, N = 8 patient cores). Staining the resulting cells for CD45 and EpCAM allowed the separation of immune, epithelial, and “double-negative” (largely stromal) cell populations (Fig. 2c). As anticipated, a benign core biopsy specimen was composed predominantly of stromal cells, while in the invasive cancer core biopsy specimens, immune and epithelial (presumably malignant) cells were the large majority of cells identified across breast cancer subtypes (Fig. 2c). Thus, the variability observed in research core biopsy specimens were concordant with the cellular compositions expected based on clinical diagnosis.

## Heterogeneity in tumor cells and immune microenvironment

**Fig. 3:**
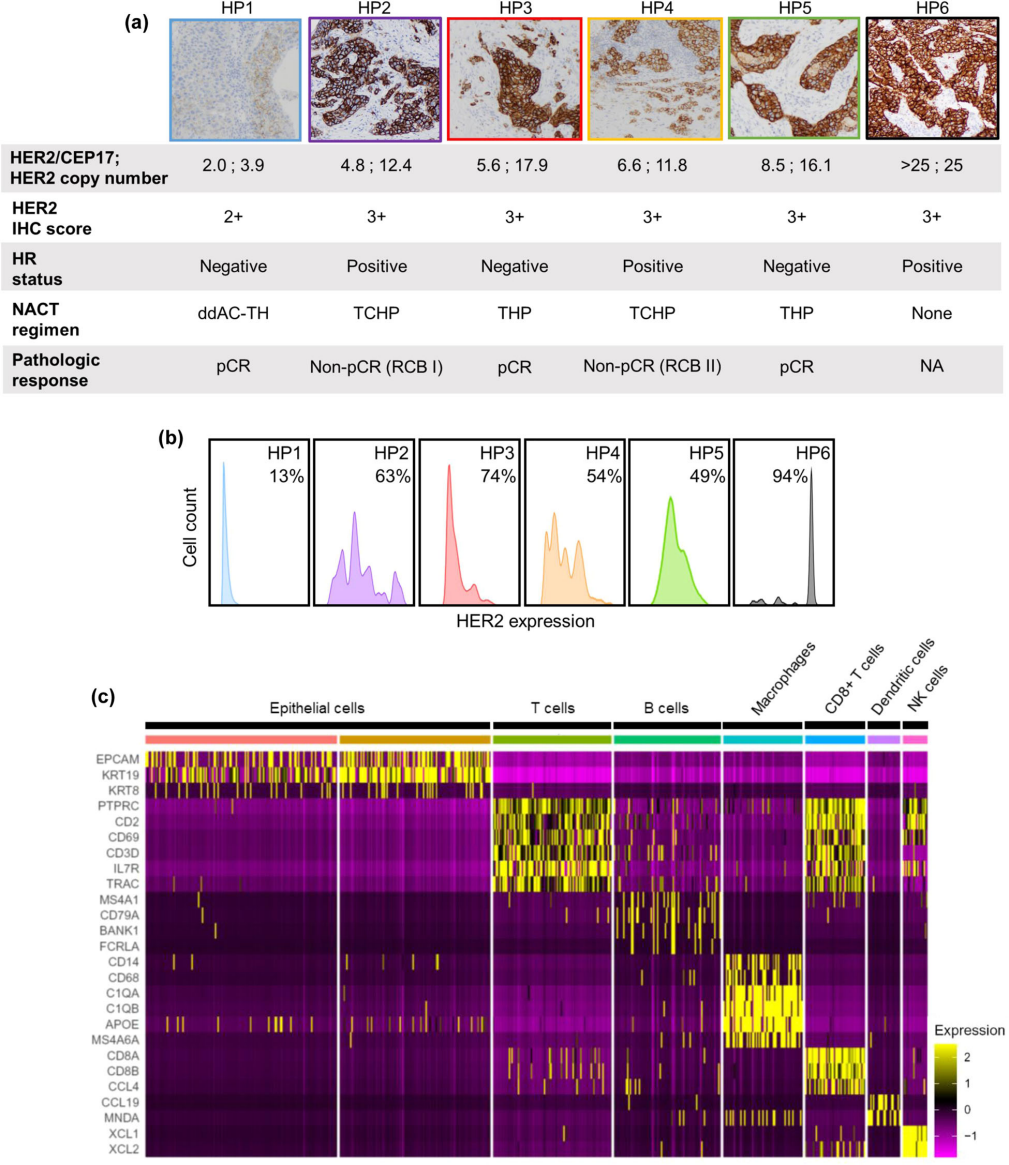
Single-cell analyses reflect extensive intratumoral heterogeneity in protein expression and tumor immune microenvironment. **a** HER2–IHC staining in clinical core biopsy specimens of HER2 + (HP) cases. Corresponding FISH (HER2/CEP17 ratio), HER2 average signal/cell (HER2 copy number), HER2 IHC scores, hormone receptor (HR) status, neoadjuvant (NACT) regimen, and pathologic response are reported on the bottom panels. ddAC–TH dose-dense doxorubicin cyclophosphamide followed by paclitaxel/trastuzumab, TCHP docetaxel carboplatin trastuzumab pertuzumab, THP paclitaxel trastuzumab pertuzumab, pCR pathologic complete response, RCB residual cancer burden^23^. Images are at 20x magnification. **b** Intertumoral and intratumoral heterogeneity of HER2 expression in HER2 + research core biopsy specimens, assessed by flow cytometry. Corresponding research core specimens in **a** were stained for EpCAM and HER2. The distribution of HER2 expression level (x-axis) reflects the extent of HER2 heterogeneity in the EpCAM + cells. Histograms are color-coded according to cases in (**a**). The percentage indicates the proportion of HER2 + cells in the EpCAM + population. **c** Heat map showing eight transcriptionally distinct clusters of cells (columns), comprising epithelial and immune cells, identified through unsupervised analysis of single-cell transcriptome data from a triple-negative biopsy specimen. Rows show the expression of key genes that define each cluster.

Emerging evidence has shown that intratumoral HER2 heterogeneity (so-called ITH-HER2), currently defined by the presence of HER2 positivity in between 5 and 50% of tumor cells or the presence of an area of tumor testing HER2-negative, is a strong predictor of treatment response in HER2-positive patients^10–14^. We thus proposed that a potentially valuable research application would be flow cytometric analysis of single cells from HER2-positive research core biopsy specimens, which can reveal the extent of intratumoral heterogeneity of HER2 expression in a detailed and quantitative manner. In a pilot study, we analyzed six such research cores by flow cytometry, co-staining for EpCAM and HER2 in order to assess tumor cell-specific HER2 expression levels. Overall HER2 positivity determined in this way was concordant with the level of HER2 amplification and with traditional histologic HER2 quantification by the immunohistochemistry (IHC) on the corresponding clinical core biopsy specimen (Fig. 3a, b). Furthermore, within the subset of HER2-positive epithelial cells, we noted a broad spectrum of HER2 expression levels that were not captured through IHC staining, although the most homogenous HER2 expression was observed in the two tumors with the highest and lowest HER2 amplification levels (Fig. 3b). Our analysis suggests that such intratumoral diversity of HER2 may be amenable to routine clinical diagnostic assessment, and it could reflect a clinically relevant aspect of this tumor driver pathway.

Finally, we performed single-cell RNA sequencing analysis on a research core biopsy specimen of triple-negative breast cancer (TNBC), an aggressive breast cancer subtype characterized by high intratumoral heterogeneity^9^. We obtained high-quality data from this specimen (587 cells, mean of 1114 genes expressed per cell) and performed unsupervised hierarchical clustering of 587 single cells and cellular gene markers specific to breast cellular types as exemplified in Fig. 3c, identifying eight transcriptionally distinct clusters, of which two were epithelial and six were immune cells (Fig. 3c and Supplementary Fig. 1a). The two epithelial clusters both exhibited luminal-like signatures but were distinguished by differential expression of the luminal epithelial marker KRT19, suggesting fate plasticity in these apparent tumor cells (Fig. 3c). Among the immune cell clusters, the T cells were predominant, a common finding in TNBC^15^. Since recent work has identified CD8 + tissue-resident memory T (T_RM_) cells as prognostically important in TNBC^16^, we investigated whether the transcriptional hallmarks of T_RM_ cell subpopulations were identifiable in this specimen. As anticipated, we found that nearly all cells harboring T_RM_ markers were in the CD8 + T cell cluster (Supplementary Fig. 1b). Furthermore, in line with the previous demonstration that most T_RM_ cells are non-mitotic, we detected few or no cells expressing mitotic T_RM_ markers. Thus, this analysis suggests the potential presence of these prognostically important immune cells, although definitive identification would be strengthened by analysis of additional cases. Altogether, 24 of 25 assessed research core biopsy specimens were validated as diagnostically concordant and amenable to quantitative laboratory analyses. Thus, our data demonstrate proof-of-principle for the systematic implementation of safe and efficient collection of breast core biopsy specimens for a variety of research applications, including various clinically relevant single-cell analyses.

The comprehensive analysis of clinically annotated tissue specimens from patients with either benign, atypical, or malignant breast findings is critical to improving the understanding of breast cancer biology and treatment. Image-guided core needle breast biopsies have become the gold standard for the diagnosis of breast cancer in most major centers. The tissues obtained with this method are used clinically to determine histopathology and thereby inform treatment decisions. However, the potential utility of such tissue collection is not limited solely to clinical decision-making. The specialized technology used to perform image-guided biopsies also allows for the collection of extra tissues that have myriad research applications.

Overall, we have demonstrated that an upfront breast research biopsy program enrolling patients presenting for initial diagnostic breast biopsy is feasible, safe, and has high rates of patient participation. Indeed, this study demonstrates that the collection of additional research biopsies did not result in a complication rate exceeding those of clinical core biopsies. It is well established that image-guided breast biopsies of non-palpable lesions are safe and maintain high diagnostic accuracy^17^. Since both the clinical and research specimens in our study were obtained using a single core biopsy, the safety we observed was not unexpected. The safety of research biopsies is largely underreported in the literature. One large single-institution study reporting on the safety of research liver biopsies compared to clinical liver biopsies found low complication rates overall, with no significant increase in complications observed for the research procedures despite the collection of several additional cores^18^.

Importantly, pathologic assessment of the research core specimens confirmed them to be highly concordant histologically with the corresponding clinical core specimens. Furthermore, the research core specimens were found overall to be of high quality both in terms of cell viability and cell number, enabling highly informative laboratory analyses. For instance, flow cytometry analysis of HER2 protein expression in a subset of HER2 + core biopsy specimens revealed substantial inter- and intratumoral heterogeneity in HER2 expression at a single-cell resolution not captured by conventional IHC staining. Recent studies have suggested that HER2 intratumoral heterogeneity may be a predictor of clinical response to the HER2-targeted therapy^10–14^. Conceivably, such heterogeneity could be particularly relevant in the setting of HER2-directed Antibody-Drug Conjugates, that may require a threshold of bound antibody to ensure delivery of sufficient payload drug to the tumor cells^10^. While further work and larger sample sets would be needed to establish its relevance, our study suggests that assessment of the HER2 at the individual cell level in this way may be practical as a potential clinical diagnostic.

In a TNBC core specimen, single-cell transcriptomic profiling showed T cells as the predominant tumor-infiltrating lymphocytes, consistent with the canonical characteristic of the TNBC microenvironment. In the same specimen, we further identified hallmarks of a distinct CD8 + T cell subpopulation that has been established to be prognostically superior to CD8 + expression alone. Thus, collectively, our data underscore the utility of such core biopsy specimens for high-content analyses with potential biological and clinical implications. Other breast tissue banking experiences support the notion that creation of such a resource encourages diverse research applications^19,20^.

Our study has several limitations. As our study focused exclusively on breast core biopsy specimens, additional studies of other organs would be required to verify if conclusions from this study can apply to other frequently biopsied anatomical sites. We also did not assess concordance between research core and clinical pathology results for all research specimens obtained. Assessment of concordance requires retrieving the fresh tissue from the tissue bank for histologic evaluation, which renders the tissue unusable for certain future applications. While the concordant results for the select number of research cores analyzed is encouraging, additional work including continuous monitoring as tissue is retrieved for use will be required to further establish the generalizability of this observed concordance. While no immediate complications were observed, it is unknown if there were delayed complications, such as biopsy site infection. An additional limitation is that this effort was a single academic center experience that depended on the engagement of the clinical breast imaging program and processes to reduce impact on clinical workflow. Likewise, there was variation over time in which types of patients were approached as more radiologists participated and the program was eventually expanded to mammographic-guided biopsies.

In summary, we have demonstrated the feasibility of our approach as a high-yield mechanism to generate a rich tissue repository, with low cost and marginal additional time commitment by patients and providers. This initiative thus provides a platform that can support a diverse variety of novel, cross-disciplinary research.

## Methods

### Identification of patients and eligibility

The potentially eligible patients were identified by a research associate on the day of scheduled biopsy through the list of scheduled breast biopsies at the Massachusetts General Hospital (MGH) breast imaging clinic in Boston, MA. Study eligibility criteria initially consisted of patients age 18 or older who were undergoing a clinical breast biopsy under US guidance, and later those undergoing biopsies under mammographic guidance were also included. For this initial cohort, patients had to have a radiographically evident mass measuring at least 0.5 cm in the longest dimension to be approached. The decision to offer a research biopsy was at the discretion of the breast radiologist performing the clinical biopsy. Signed informed consent for research core collection was obtained in the procedure room by the research associate immediately following the clinical consent process by the procedural radiologist and prior to the clinical biopsy. Identifying and consenting patients to the research study did not require any modifications to existing clinical scheduling procedures.

### Acquisition of clinical and research biopsies

According to institutional practice, 2–5 cores (for US-guided biopsies) or 6–12 cores (for mammographic-guided biopsies) per lesion were obtained at the discretion of the breast radiologist performing the clinical biopsy after the administration of local anesthesia (lidocaine). US guided biopsies utilized a 12 or 14 G spring loaded core-needle biopsy device and mammographic-guided biopsies utilized a 9 G vacuum-assisted biopsy device. MRI biopsies were not included because it is not possible to confirm adequate tissue for clinical diagnosis such that remaining tissue could be set aside for research purposes. These clinical cores were sent to pathology for standard processing and evaluation. For patients who consented to the research protocol, following the collection of all necessary routine clinical specimens, the breast radiologist obtained at most two additional cores per lesion, using the same biopsy needle as was used for obtaining the prior clinical specimens. The additional research cores were placed on a Telfa pad and immediately handed off to the research associate for processing. In the case of US-guided biopsies, the first research core was immediately flash frozen and stored on dry ice to preserve high molecular weight DNA and RNA, while the second core was cryopreserved to allow viable cell recovery. In the case of mammographic-guided biopsies, both cores were cryopreserved. The workflow is depicted in Fig. 1.

### Database

All consented patients are entered into a secure database in REDCap (Research Electronic Data Capture), a secure, web-based software platform^21,22^. Medical records of the participating patients are reviewed periodically to collect the data on patient demographics, biopsy results, and if applicable, tumor characteristics, treatment characteristics, and long-term outcomes.

### Assessment of complications

Complication rates were determined by a review of the breast imaging database (Magview, Fulton, MD), which tracks all procedures and the occurrence of any immediate complications noted at the time of the biopsy. Routine bleeding, bruising, or pain at the biopsy site is not considered a complication. Examples of possible immediate complications include severe vasovagal reaction, marked bleeding, or pneumothorax.

### Generation of single-cell suspensions

Two research core biopsy specimens were typically obtained from each consenting patient. One core was snap-frozen on dry ice and kept at −80° C for long-term storage. The second core was used by laboratory-based researchers to generate a single-cell suspension. To this end, the core was minced into smaller pieces using a scalpel. The minced tissue was then digested enzymatically using a human tumor dissociation kit (Miltenyi Biotec) and mechanically on a rocker in an incubator at 37° C. The whole digestion took approximately 1–2 h; the solution was then centrifuged at 450 G for 5 min. The supernatant was removed, and the pellet was resuspended in 5% dimethyl sulfoxide in fetal bovine serum (Sigma). The suspension was then stored at − 80° C overnight in a cryogenic box before long-term storage in a liquid nitrogen tank.

### Immunohistochemistry

Core biopsy specimens were fixed in 4% buffered formalin for 24 h and then transferred to 70% ethanol. Sections of 5-µm thickness were acquired from formalin-fixed, paraffin-embedded biopsies and stained using standard protocols.

### Flow cytometry

The cryopreserved suspension was rapidly thawed. Cells were first filtered with a 70 µm filter, blocked with blocking buffer, and then stained with the appropriate conjugated antibodies for 30 min in the dark at 4° C. In some cases, cells were fixed using 10% formalin at 37° C prior to antibody staining. In unfixed cases, cells were counterstained with DAPI for viability. Antibodies were washed off with phosphate-buffered saline prior to flow cytometric analysis. Antibodies used were EpCAM/CD326 (BioLegend #324208, 1:20 dilution), CD45 (Biolegend #304034, 1:20 dilution), and HER2/ErbB2 (Cell Signaling #98710, 1:20 dilution). Gating strategies for the flow cytometric data are shown in Supplementary Fig. 2.

### Single-cell RNA sequencing and data processing

Droplet-based single-cell RNA sequencing was performed on single-cell suspension using the Chromium Single Cell 3' v3 Library, Gel Bead and Chip Kit (10X Genomics), according to the manufacturer’s instruction. The library was sequenced on the Illumina HiSeq2500 platform with paired-end sequencing. Sample demultiplexing, reference mapping, barcode processing and gene counting were performed using Cell Ranger 4.0.0 (10X Genomics). Reads were aligned to the GRCh38 human reference genome. Genes expressed in at least 10 cells (yielding 10,569 genes), as well as cells with at least 100 genes expressed and < 20% mitochondria (587 cells) were retained. The data was preprocessed with SCTransform as implemented in Seurat 4.0.1, and normalized data was used as input for PCA, followed by dimensionality reduction on the PCA space with Uniform Manifold Approximation and Projection (UMAP). All functions were used with default parameters.

### Ethics

This study received the institutional review board (IRB) approval from the Mass General Brigham Human Research Committee.

### Reporting Summary

Further information on research design is available in the Nature Research Reporting Summary linked to this article.

## Supplementary information

Supplementary Information

Reporting Summary

## Data Availability

Single-cell RNA sequencing data in this publication have been deposited in the NCBI’s Gene Expression Omnibus and are accessible through GEO Series accession number GSE177482. Other data that support the findings of this study are available from the corresponding author upon reasonable request.
