# Supplementary material for: Systematic tissue collection during clinical breast biopsy is feasible, safe and enables high-content translational analyses
Source: NPJ Precis Oncol. 2021 Sep 21;5:85. doi: 10.1038/s41698-021-00224-w (PMC8455592; doi:10.1038/s41698-021-00224-w)
Supplement: Supplementary file 1 — Supplementary Information [file 41698_2021_224_MOESM1_ESM.pdf]

SUPPLEMENTARY FIGURES

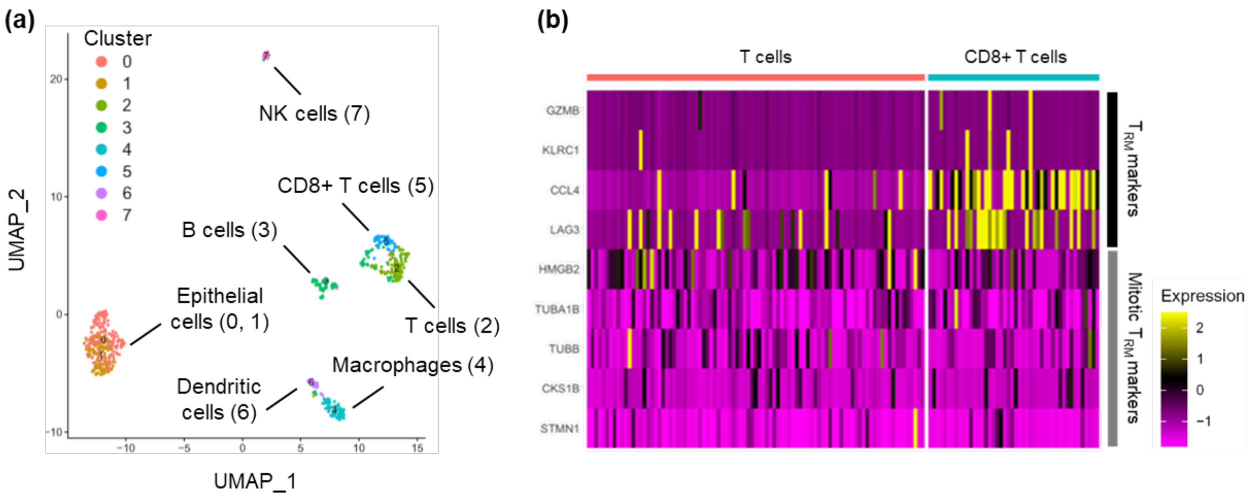

Supplementary Figure 1. Single-cell transcriptomic analysis shows cellular diversity in a research core biopsy specimen.

(a) High-dimensional uniform manifold approximation and projection (UMAP) clustering of single-cell transcriptomic data from a triple-negative breast core biopsy specimen shows a diverse composition of epithelial, lymphoid and myeloid cells.

(b) Heatmap of single-cell transcriptome of all T cells identified in the triple-negative breast core biopsy specimen. T cells positive for non-mitotic T_{RM} markers (top) were enriched in the CD8+ T cell cluster, while few or none of them were positive for mitotic T_{RM} markers (bottom). T_{RM} markers were derived from a previous publication (Savas P et al., 2018).

See also Figure 3C.

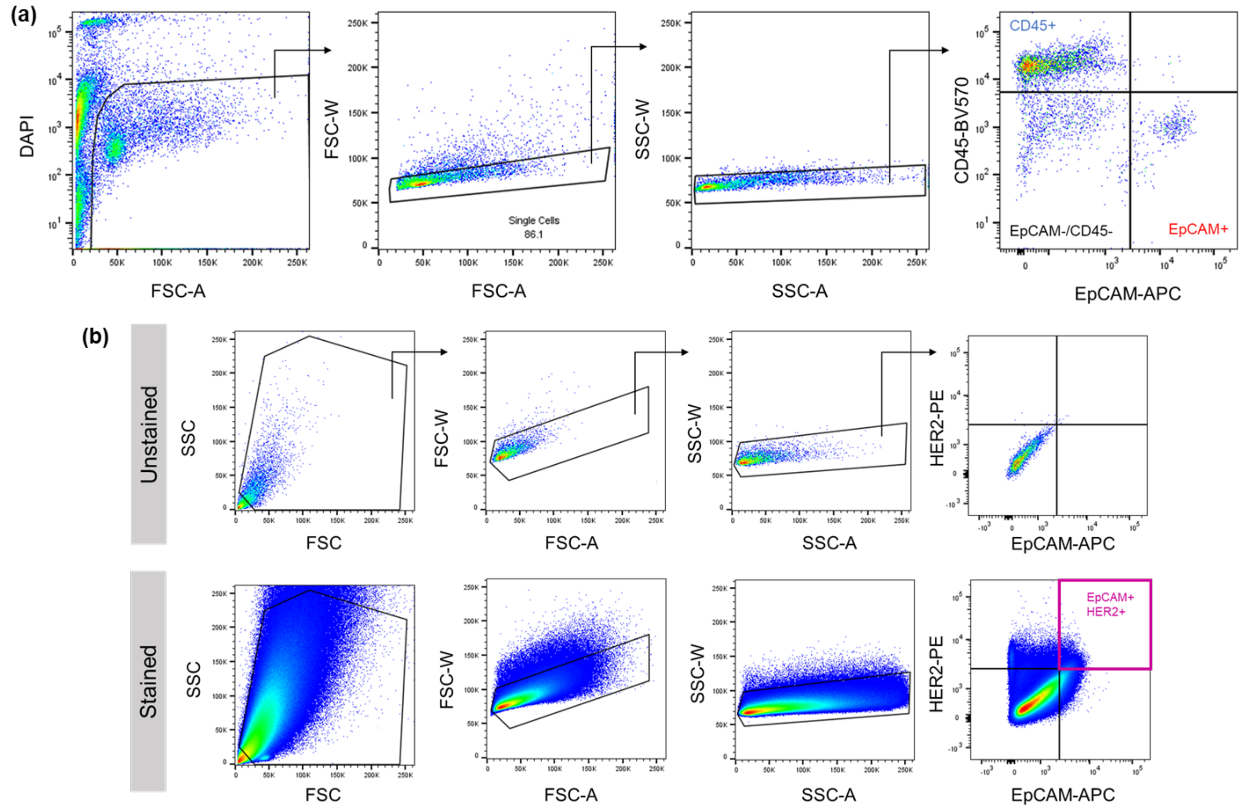

Supplementary Figure 2. Gating strategies were used to define specific cell populations in research core biopsy specimens.

(a) Gating strategy used to identify viable (DAPI-negative) EpCAM-positive, CD45-positive or double-negative cells. See also Figure 2c.

(b) Gating strategy used to identify formalin-fixed EpCAM/HER2-positive cells. See also Figure 3b.

SUPPLEMENTARY TABLE

Specimen<br>-Clinical specimen<br>-Research specimen	Primary pathologic diagnosis	Grade	Cellularity
1	IDC<br>IDC	1<br>1	< 5%<br>< 5%
2	IDC<br>IDC	1<br>1	10%<br>< 1%
3	Fibroadenoma<br>Fibroadenoma	N/A<br>N/A	N/A<br>N/A
4	IDC<br>IDC	2-3<br>2	20%<br>20%
5	ILC<br>ILC	2-3<br>2	30%<br>30%
6	IDC<br>IDC	3<br>3	20%<br>30%
7	IDC<br>IDC	3<br>3	60%<br>10%
8	ILC<br>ILC	2<br>2	30%<br>30%
9	IDC<br>Non-diagnostic, debris	2<br>N/A	50%<br>N/A
10	IDC<br>IDC	2<br>2	40%<br>40%

Legend: IDC, invasive ductal carcinoma; ILC, invasive lobular carcinoma.

Supplementary Table 1. Concordance between clinical and research biopsy specimens.
