# Supplementary material for: Systematic tissue collection during clinical breast biopsy is feasible, safe and enables high-content translational analyses
Source: NPJ Precis Oncol. 2021 Sep 21;5:85. doi: 10.1038/s41698-021-00224-w (PMC8455592; doi:10.1038/s41698-021-00224-w)
Supplement: Supplementary file 2 — Reporting Summary [file 41698_2021_224_MOESM2_ESM.pdf]

nature research

Corresponding author(s): DBPR NPJPRECISIONONCOLOGY-01226

Last updated by author(s): Jun 23, 2021

Reporting Summary

Nature Research wishes to improve the reproducibility of the work that we publish. This form provides structure for consistency and transparency in reporting. For further information on Nature Research policies, see our Editorial Policies and the Editorial Policy Checklist.

Statistics

For all statistical analyses, confirm that the following items are present in the figure legend, table legend, main text, or Methods section.

n/a	Confirmed	
☐	☒	The exact sample size (*n*) for each experimental group/condition, given as a discrete number and unit of measurement
☐	☒	A statement on whether measurements were taken from distinct samples or whether the same sample was measured repeatedly
☒	☐	The statistical test(s) used AND whether they are one- or two-sided<br>*Only common tests should be described solely by name; describe more complex techniques in the Methods section.*
☒	☐	A description of all covariates tested
☒	☐	A description of any assumptions or corrections, such as tests of normality and adjustment for multiple comparisons
☒	☐	A full description of the statistical parameters including central tendency (e.g. means) or other basic estimates (e.g. regression coefficient) AND variation (e.g. standard deviation) or associated estimates of uncertainty (e.g. confidence intervals)
☒	☐	For null hypothesis testing, the test statistic (e.g. *F*, *t*, *r*) with confidence intervals, effect sizes, degrees of freedom and *P* value noted<br>*Give P values as exact values whenever suitable.*
☒	☐	For Bayesian analysis, information on the choice of priors and Markov chain Monte Carlo settings
☒	☐	For hierarchical and complex designs, identification of the appropriate level for tests and full reporting of outcomes
☒	☐	Estimates of effect sizes (e.g. Cohen's *d*, Pearson's *r*), indicating how they were calculated

Our web collection on statistics for biologists contains articles on many of the points above.

Software and code

Policy information about availability of computer code

Data collection	Data collection was carried out using BD LSRII flow cytometer and 10X single-cell sequencing platform.
Data analysis	Data analysis was carried out using FlowJo V10 and Seurat 4.0.1.

For manuscripts utilizing custom algorithms or software that are central to the research but not yet described in published literature, software must be made available to editors and reviewers. We strongly encourage code deposition in a community repository (e.g. GitHub). See the Nature Research guidelines for submitting code & software for further information.

Data

Policy information about availability of data

All manuscripts must include a data availability statement. This statement should provide the following information, where applicable:

- Accession codes, unique identifiers, or web links for publicly available datasets
- A list of figures that have associated raw data
- A description of any restrictions on data availability

Single cell RNA sequencing data in this publication have been deposited in NCBI's Gene Expression Omnibus and are accessible through GEO Series accession number GSE177482 (https://www.ncbi.nlm.nih.gov/geo/query/acc.cgi?acc=GSE177482). Other data that support the findings of this study are available from the corresponding author upon reasonable request.

Field-specific reporting

Please select the one below that is the best fit for your research. If you are not sure, read the appropriate sections before making your selection.

☒ Life sciences ☐ Behavioural & social sciences ☐ Ecological, evolutionary & environmental sciences

For a reference copy of the document with all sections, see nature.com/documents/nr-reporting-summary-flat.pdf

Life sciences study design

All studies must disclose on these points even when the disclosure is negative.

Sample size	Number of samples used was reflected accordingly in the manuscript.
Data exclusions	No data were excluded.
Replication	N/A
Randomization	N/A
Blinding	N/A

Reporting for specific materials, systems and methods

We require information from authors about some types of materials, experimental systems and methods used in many studies. Here, indicate whether each material, system or method listed is relevant to your study. If you are not sure if a list item applies to your research, read the appropriate section before selecting a response.

Materials & experimental systems

n/a	Involved in the study	
☐	☒	Antibodies
☒	☐	Eukaryotic cell lines
☒	☐	Palaeontology and archaeology
☒	☐	Animals and other organisms
☐	☒	Human research participants
☒	☐	Clinical data
☒	☐	Dual use research of concern

Methods

n/a	Involved in the study	
☒	☐	ChIP-seq
☐	☒	Flow cytometry
☒	☐	MRI-based neuroimaging

Antibodies

Antibodies used	EpCAM/CD326 (Biolegend #324208), CD45 (Biolegend #304034), HER2/ErbB2 (Cell Signaling #98710)
Validation	These commercial antibodies were used in accordance with manufacturer's recommendations.

Human research participants

Policy information about studies involving human research participants

Population characteristics	Study eligibility criteria initially consisted of patients age 18 or older who were undergoing a clinical breast biopsy under ultrasound guidance, and later those undergoing biopsies under mammographic guidance were also included. For this initial cohort, patients had to have a radiographically-evident mass measuring at least 0.5 cm in the longest dimension to be approached.
Recruitment	Potentially eligible patients were identified by a research associate on the day of scheduled biopsy through the list of scheduled breast biopsies at the Massachusetts General Hospital (MGH) breast imaging clinic in Boston, MA. Study eligibility criteria initially consisted of patients age 18 or older who were undergoing a clinical breast biopsy under ultrasound guidance, and later those undergoing biopsies under mammographic guidance were also included. For this initial cohort, patients had to have a radiographically-evident mass measuring at least 0.5 cm in the longest dimension to be approached. The decision to offer a research biopsy was at the discretion of the breast radiologist performing the clinical biopsy. Signed informed consent for research core collection was obtained in the procedure room by the research associate immediately following the clinical consent process by the procedural radiologist and prior to the clinical biopsy.
Ethics oversight	This study received institutional review board (IRB) approval from the Mass General Brigham Human Research Committee.

Note that full information on the approval of the study protocol must also be provided in the manuscript.

Flow Cytometry

Plots

Confirm that:

- [x] The axis labels state the marker and fluorochrome used (e.g. CD4-FITC).
- [x] The axis scales are clearly visible. Include numbers along axes only for bottom left plot of group (a 'group' is an analysis of identical markers).
- [ ] All plots are contour plots with outliers or pseudocolor plots.
- [x] A numerical value for number of cells or percentage (with statistics) is provided.

Methodology

Sample preparation	Cells were filtered with a 70 µm filter, blocked with blocking buffer and then stained with appropriate conjugated antibodies for 30 minutes in the dark at 4 degreess Celsius. In some cases, cells were fixed using 10% formalin at 37 degrees Celsius prior to antibody staining. In unfixed cases, cells were counterstained with DAPI for viability.
Instrument	BD LSRII flow cytometer
Software	FlowJo V10
Cell population abundance	Cell population was determined based on indicated markers, and the aduncance was reflected in the figure as percentage.
Gating strategy	Gating of positive population was done based on unstained sample.

- [ ] Tick this box to confirm that a figure exemplifying the gating strategy is provided in the Supplementary Information.
